# Immediate anterior open reduction and plate fixation in the management of lower cervical dislocation with facet interlocking

**DOI:** 10.1038/s41598-018-37742-w

**Published:** 2019-02-04

**Authors:** Yuwei Li, Peng Zhou, Wei Cui, Cheng Li, Wei Xiao, Yan Wen, Haoran Wang, Haijiao Wang

**Affiliations:** 1 Department of Spine Surgery, Luohe Central Hospital, Luohe Medical College, Luohe, 462000 People’s Republic of China; 2 Department of Cardiology Ward 2, Luohe Central Hospital, Luohe Medical College, Luohe, 462000 People’s Republic of China

## Abstract

Lower cervical dislocation with facet interlocking is one of the most drastic injuries to the cervical spine. The early reduction is thought critical in preventing progressive secondary spinal cord injury. The authors report a new surgical procedure in the management of lower cervical dislocation with facet interlocking. A total of twenty-one cases received immediate single-staged anterior open reduction, realignment and plate fixation under general anesthesia. After the procedures, most cases exhibited improved neurological function. All patients showed stable fusion at 1-year follow-up. Loss of spinal alignment or kyphotic deformity was not found in any case. Hardware failure including screw loosening or penetrating was not observed. In conclusion, the immediate anterior open reduction and plate fixation is a safe and effective procedure in the management of lower cervical dislocation with facet interlocking.

## Introduction

Cervical dislocation with facet interlocking is one of the most drastic injuries to the cervical spine. The clinical features including dislocation of the facet joints as well as neurological dysfunction are caused by primary and secondary injury^1,2^. The goals of treatment of any spinal injury include neurological recovery and spinal segment reconstruction. Although a few studies have reported the treatment procedures of this special clinical situation^3–5^, there are still controversies about the methods of reduction, the timing for surgery, and the modes of stabilization^6,7^. Currently, realignment by closed traction, followed by anterior, posterior or combined fixation/fusion has been the most common choice, and open reduction has been performed only after failed closed reduction^4,8,9^. While the use of axial traction to reduce dislocated joints and realign the fractured segment remains the mainstay of acute management, early operative treatment has gained increasing acceptance in recent years^10–12^. The current study aims to take the surgical procedure by immediate reduction as well as reconstruction of the cervical sequence and stability through an anterior surgical procedure.

## Methods

### Study design

This study is a single center case series which was conducted in Luohe Central Hospital, which was specialized in the management of spinal cord diseases. The study is conducted according to the guideline for case series^13^. Patients were enrolled from January 2006 to July 2016. The inclusion criteria were: (1) age >16; (2) lower cervical dislocation with facet interlocking confirmed by X-ray/MRI/CT. The exclusion criteria were: (1) neurological or cognitive impairment precluding reliable neurological evaluation; (2) penetration injury; (3) with vertebral/vertebral endplate fracture; (4) with serious osteoporosis; (5) presented in a life-threatening situation with an immediate surgical contraindication. During the research period all patients were assessed for suitability according to the inclusion and exclusion criteria. The study protocol was approved by the ethnic board of Luohe Central Hospital. We stated that informed consent was obtained from all participants and/or their legal guardians.

### Management procedure

After admission to the hospital, neurological examination was carried out according to the Frankel classification. To illustrate the extent of the fracture and to determine the adequate surgical procedure, all the patients received both anteroposterior and lateral X-rays as well as a computed tomography (CT) and magnetic resonance imaging (MRI) according to standard protocol.

After the evaluation, the qualified patients will be chosen to take the appropriate surgical procedure to reduce the compression of the spinal cord and reconstruct the cervical sequence and stability.

We adopted an anterior surgical procedure. Under the general anesthesia with intubation, patients were placed at a supine position, with the fixation of forehead. A standard right-sided transverse incision was used. Vertebral levels were identified with intraoperative C-arm fluoroscopic visualization. Complete discectomies were accomplished at the involved level, and protruded disc fragments or small bone fragments compressing the medulla were carefully removed. For unilateral facet interlocking, the distractor screw holes were drilled in the locked side of the vertebral body at the medial margin of the musculus longus colli. For bilateral facet interlocking, the holes were drilled at the median line of the vertebral body. After this, the interspace was gradually widened to about 5–7 millimeter by carefully operating the Caspar distractor (Zhangjiakou Sanxing Medical Instruments Co., Ltd, Zhangjiakou). Thereafter, a periosteal elevator (Zhangjiakou Sanxing Medical Instruments Co., Ltd, Zhangjiakou) was inserted at the disc space to resolve facet locking by a slight distracting force. Finally, the articular gap was filled with allograft bone to facilitate fusion, and the stabilization was carried out with the use of zero-profile plate (Xiamen Dabo Medical Instruments Co., Ltd, Xiamen), which ensured the fusion between the adjacent vertebrae above and below the injured vertebral body.

After surgery, all the patients were treated and rehabilitated in a timely manner as usual. Neurological status was recorded at initial presentation, preoperatively, and at time of discharge from the hospital or transfer to a rehabilitation unit. Neurological function was recorded as Frankel grade. The Frankel Grade was recorded as Levels 1 to 5, corresponding to Grades A to E, respectively. All the medical treatments are in accordance with the Chinese Expert consensus on Evaluation, Treatment and Rehabilitation of Traumatic Spinal Cord Injury^14^.

## Results

### Participants

**Figure 1:**
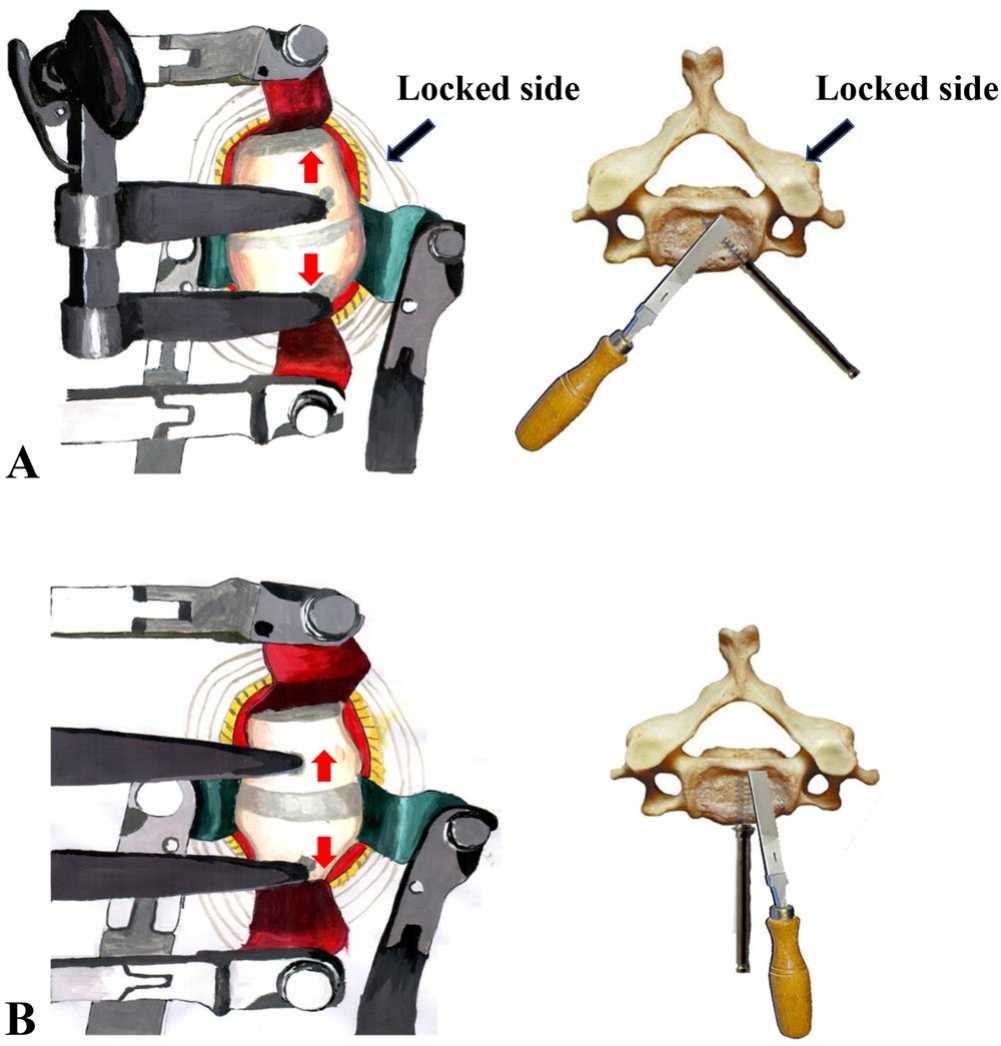
Schematic diagrams to illustrate the surgery procedure. (**A**) For unilateral facet interlocking, the distractor screw holes are drilled in the locked side of the vertebral body at the medial margin of the longus colli; the interspace is gradually widened to about 5–7 millimeter by carefully operating the Caspar distractor mechanism; and a periosteal elevator is inserted at the disc space to resolve facet interlocking by a slight distracting force. (**B**) For bilateral facet interlocking, the holes are drilled at the median line of the vertebral body; the interspace is gradually widened to about 5–7 millimeter by carefully operating the Caspar distractor mechanism; and a periosteal elevator is inserted at the disc space to resolve facet locking by a slight distracting force.

**Figure 2:**
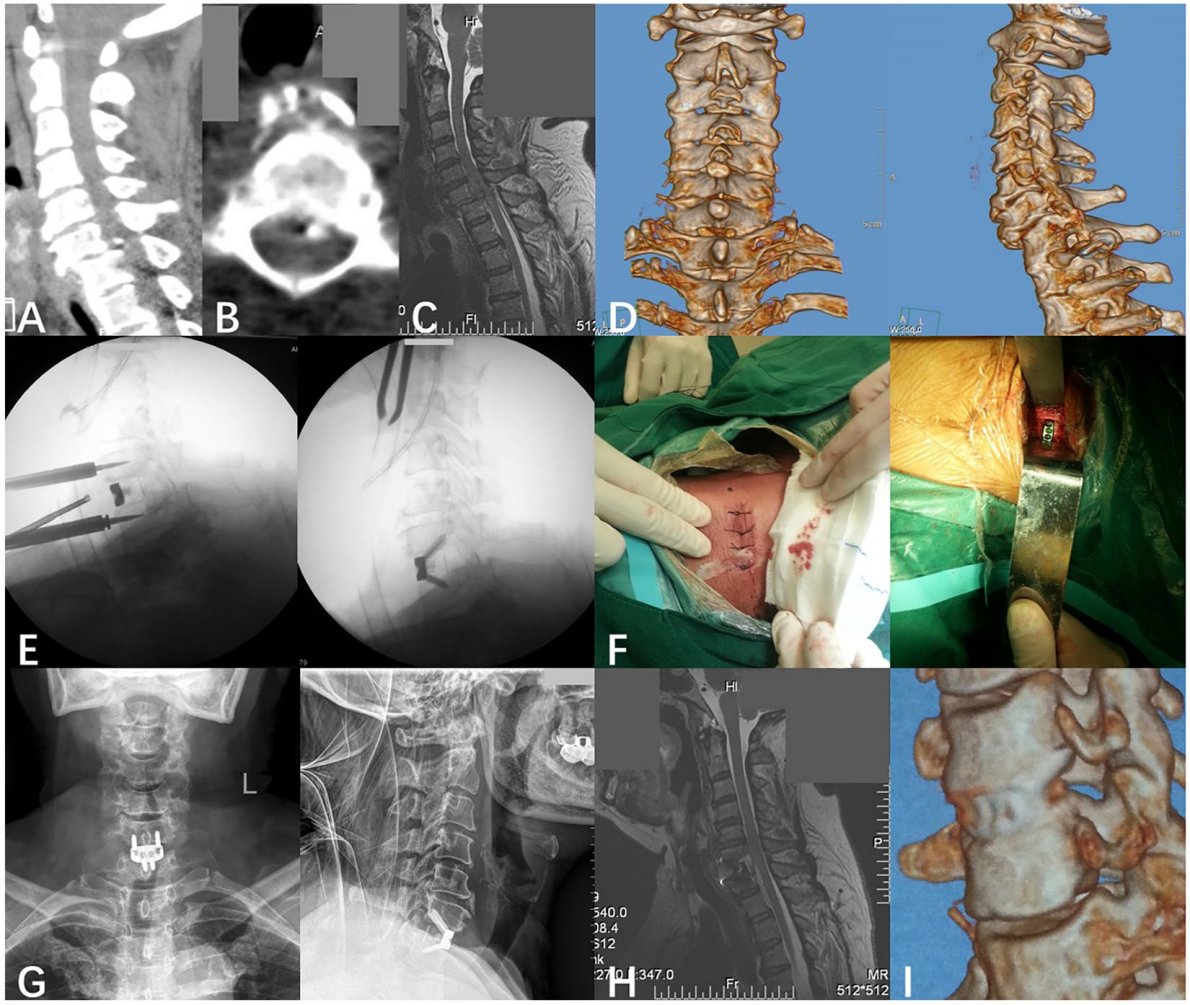
Protocol patient with C6/7 dislocation and right-side facet interlocking. (**A**) At presentation, sagittal position CT indicates C6/7 dislocation, and a small bone fragment presents at the posterior margin of the vertebral body of C6. (**B**) Horizontal position CT reveals a small bone fragment presents at the posterior margin of the vertebral body of C6. (**C**) Sagittal position MRI reveals C6/7 dislocation, disc herniation and compression of the spinal cord. (**D**) Stereoscopic reconstructed CT reveals C6/7 dislocation and facet interlocking at the right-side of C6. (**E**) Intraoperative C-arm fluoroscopic visualization. (**F**) Operative incision. (**G**) The anteroposterior and lateral view of cervical spine radiography after surgery. (**H**) Two weeks after the surgery, the MRI indicates the cervical sequence and stability was reconstructed, and the compression of the spinal cord was released completely. (**I**) Three months after the surgery, the reconstructed CT reveals complete postoperative realignment and fusion was achieved.

During the study period, 21 patients (14 males) fulfilled the inclusion criteria. Thirteen patients sustained their injuries in traffic accidents, 6 patients were injured in falls, and 2 patients were injured by falling objects. Dislocation occurred at C3/4 in 2 patients, C4/5 in 4 patients, C5/6 in 4 patients and C6/7 in 11 patients. In 7 patients the facet interlocking was unilateral; in 14 patients it was bilateral. Neurological symptoms were presented in all cases, although with different degrees of severity. The detailed characteristics of the included patients are listed in Table 1. Surgery procedure is illustrated in Fig. 1. And the surgery images of a protocol patient are presented in Fig. 2.

**Table 1:** Characteristics of the included patients.

Case no.	Age	Gender	Level	Frankel pre-operation	Frankel post operation	Facet interlocking	Causes of injury	Injury to surgery time (hour)	Follow-up time (month)
1	25	Male	C4/5	B	D	Unilateral	Traffic accident	10	19
2	67	Male	C6/7	B	E	Bilateral	Falling injury	17	17
3	22	Male	C4/5	B	D	Unilateral	Traffic accident	18	15
4	28	Male	C6/7	B	B	Bilateral	Traffic accident	22	18
5	48	Male	C3/4	B	D	Bilateral	Falling injury	18	16
6	23	Male	C3/4	B	C	Unilateral	Traffic accident	21	15
7	40	Male	C6/7	B	C	Bilateral	Traffic accident	19	18
8	57	Female	C6/7	B	C	Unilateral	Traffic accident	12	14
9	45	Male	C5/6	A	C	Bilateral	Falling injury	16	15
10	60	Male	C6/7	B	B	Bilateral	Traffic accident	19	16
11	49	Female	C5/6	C	C	Unilateral	Traffic accident	17	17
12	54	Female	C6/7	E	E	Bilateral	Traffic accident	11	16
13	48	Female	C6/7	A	D	Unilateral	Traffic accident	14	14
14	37	Female	C4/5	A	A	Bilateral	Traffic accident	18	17
15	23	Male	C4/5	C	E	Bilateral	Traffic accident	19	15
16	38	Male	C6/7	C	D	Unilateral	Falling injury	22	16
17	32	Female	C5/6	C	E	Bilateral	Falling objects accident	18	21
18	51	Male	C6/7	D	D	Bilateral	Traffic accident	23	17
19	64	Male	C6/7	D	E	Bilateral	Falling injury	17	25
20	43	Female	C5/6	D	E	Bilateral	Falling injury	25	37
21	47	Male	C6/7	D	E	Bilateral	Falling objects accident	15	21
Median value	45							18	17

### Outcomes and follow-up

Patients were operated on timely after admission to the hospital and no intraoperative complications were observed. Cerebrospinal fluid leakage occurred in 2 cases which subsided with conservative treatment. During the follow-up period, all 21 patients showed stable fusion at a minimum of 1-year follow-up, assessed by lateral radiographs as isodense bone bridging across the endplates. Loss of spinal alignment or kyphotic deformity was not found in any case. Hardware failure including screw loosening or penetrating was not observed. At the 1-year follow-up, none of the patients complained of relevant neck pain.

**Figure 3:**
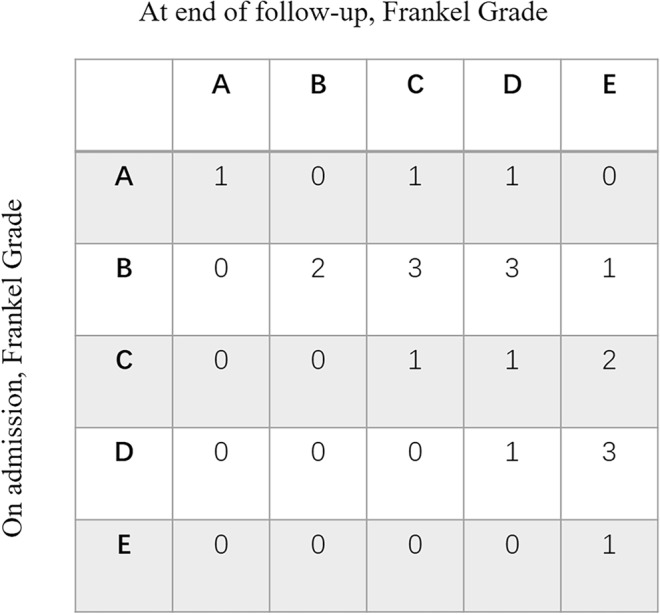
The neurological status on admission and at end of follow-up.

The neurological status on admission and by the end of follow-up is listed in Fig. 3. During the course of the follow-up, 15 patients (71.4%) exhibited improved neurological function, and deterioration of neurological function was not observed.

## Discussion

### Main results

The current study reported a new surgical procedure in the management of lower cervical dislocation with facet interlocking, which combined the mostly important two steps: the reliving the spinal compression and the reconstruction of the cervical sequence and stability. The results indicated that the neurological function was greatly improved in the majority patients while no deterioration of neurological function was observed. Besides, complete postoperative realignment and fusion was achieved in all patients and maintained throughout the follow-up period.

### In relevance to other studies

Patients suffering cervical dislocation are at high risk of multiple systemic complications including infections of pulmonary or urinary tract, musculoskeletal contractions and deep vein thrombosis. Great efforts have made to improve the prognosis of the disease and to promote the rehabilitation of patients. Despite the great progressions have been made in recent years, the optimal medical and surgical treatment procedure still remains controversial.

One of the controversies centers on how to perform the reduction. It has been well documented that the nerve injury involves both primary and secondary injury mechanisms^15^. The primary injury, usually caused by rapid spinal cord compression and contusion, initiates a signaling cascade of down-stream events collectively known as secondary injury. Because damage in the primary phase cannot be prevented, all approaches focus on restricting damage in the secondary phase of the injury^16^, and reduction has been deemed as the most effective measure to prevent progressive secondary injury^17^. While closed reduction remains the mainstay of acute management^18^, the drawbacks of this method have been well documented^19^. Closed reduction was performed through cervical traction under local anesthesia, along with immobilization in a Halo thoracic brace. The force was increased gradually until the spinal canal has been restored to at least two-thirds its normal sagittal diameter, followed by an open surgery to reduce fracture-dislocation or to remove soft-tissue compression^8^. The closed reduction procedure may cost 1 hour or more, which precludes an immediate reduction. Furthermore, to ensure the safety of the reduction process, surgeons must confirm that there are no occupying lesions such as disc-material and bony fragments which can cause cord damage during the reduction; the reduction process should be monitored intensively; and a closed reduction should not be attempted on an obtunded patient in most circumstances. On the contrary, all the drawbacks mentioned above could be resolved by the immediate open reduction surgery.

Another controversy centers on which approach should be chosen^20–22^. The anterior approach is generally the best choice when reduction of the neural elements is required^23,24^, it has been reported to result in high fusion rates, good clinical outcomes and low rates of fixation failure^22,25,26^. The main deficit of the anterior approach is the mechanically instability, which has led to the clinical recommendation for additional posterior fixation or external immobilization^27^. While combined anterior and posterior fixation results in much better stability, patients may expose to increased risk of iatrogenic injury due to the longer operation time, the need to change the patient’s position, and the complexity of the operation. An anterior approach along with plate stabilization overcomes all the deficits by limiting surgical trauma, reducing operation time and no need to change body position during the procedure. Besides, immediate postoperative stability also allows for earlier mobilization and subsequent rehabilitation.

The safety of the surgical procedure was also documented in the current study. Serious complications including infection, reaction to foreign bodies and loosening screws were not experienced. The most feared complication, penetration of the dura and spinal cord damage by drilling or screw placement, was not encountered. There was no one case of worsening neurological function after the procedure. An ultimately stable fusion position was achieved in every case. Additionally, the Caspar distractor has made the procedure simpler and safer, by providing better visualization of the disc space, superior distraction and alignment. Nevertheless, fixation failure has been reported by previous studies with similar surgical procedure^25,26^. For example, Johnson *et al*. reported an overall radiographic failure rate of 13% in 87 patients with unilateral and bilateral facet fracture subluxations who were treated by anterior cervical discectomy, fusion, and plating. The failure is strongly correlated with fractures of the facets and fractures of the superior endplate of the lower vertebra. Anissipour *et al*. reported a treatment failure rate of 8% in 36 patients. According to their study, endplate fractures of the inferior level in jumped facets appears to be a major risk factor of fixation failure. However, in the series of the current study, according to the exclusion criteria, we had excluded those patients with facet or endplate fracture. This may explain the 100% fusion rate, no incidence of hardware failure or subsidence after surgery. Still, surgeons should be required to be specifically trained to perform this procedure safely.

### Limitations

When interpreting the results of the current study, several limitations need to be acknowledged. First, while potential confounding factors was minimized by the small number of patients in a single center, it is difficult to extrapolate our results to other hospitals. Second, the current study is designed as an uncontrolled case series and lacks a contemporaneous internal control group. In the future, researchers should organize prospective multi-center trials to deliver valuable data regarding the optimal management procedure with convincing results.

## Conclusions

The immediate anterior open reduction and plate fixation is a safe and effective procedure in the management of lower cervical dislocation with facet interlocking. The reduction is done immediately with the Caspar distractor under visual inspection, which allow complete removal of bone fragments and disc material extruded into the spinal canal from the fracture-dislocations. Facet interlocking is resolved by a slight distracting force with the use of a periosteal elevator. Stable fusion with the use of zero-profile plate was observed in all patients of the current study. Therefore, we prefer this technique over others in the management of lower cervical dislocation with facet interlocking. Future studies with a prospective design and larger sample size are needed to further validate the anterior-only surgical approach in the management of lower cervical dislocation and to explore the most appropriate surgical method for those with facet or endplate fracture.
